# Supplementary figures and images for: New Transcriptomic Biomarkers of 5-Fluorouracil Resistance
Source: Int J Mol Sci. 2023 Jan 12;24(2):1508. doi: 10.3390/ijms24021508 (PMC9867124; doi:10.3390/ijms24021508)

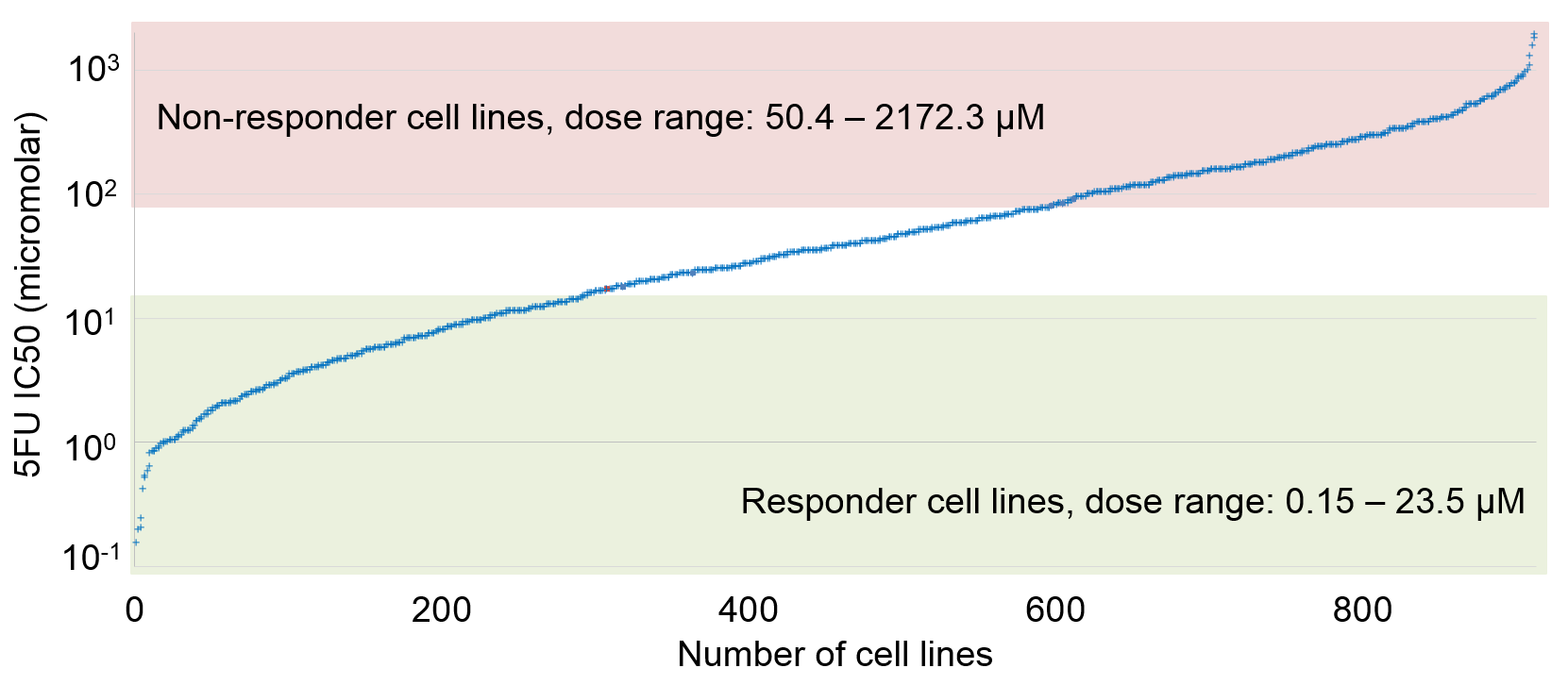

Supplement: Supplementary file 1 [file ijms-24-01508-s001.zip › Supplemental_Figure_S1.tif]
